# A conditional null allele of *Dync1h1* enables targeted analyses of dynein roles in neuronal length sensing

**DOI:** 10.1242/jcs.260220

**Published:** 2022-10-31

**Authors:** Agostina Di Pizio, Letizia Marvaldi, Marie-Christine Birling, Nataliya Okladnikov, Luc Dupuis, Mike Fainzilber, Ida Rishal

**Affiliations:** ^1^ Departments of Biomolecular Sciences and Molecular Neuroscience, Weizmann Institute of Science, Rehovot 7610001, Israel; ^2^ Department of Neuroscience “Rita Levi Montalcini”, Neuroscience Institute Cavalieri Ottolenghi, University of Turin, Orbassano 10043, Italy; ^3^ PHENOMIN-Institut Clinique de la Souris, Université de Strasbourg, Illkirch 67404, France; ^4^ INSERM U1118, Université de Strasbourg, 67085 Strasbourg, France

**Keywords:** Dynein heavy chain, Axonal transport, Length sensing, Cell size, Neurodegeneration

## Abstract

Size homeostasis is a fundamental process in biology and is particularly important for large cells such as neurons. We previously proposed a motor-dependent length-sensing mechanism wherein reductions in microtubule motor levels would be expected to accelerate neuronal growth, and validated this prediction in dynein heavy chain 1 Loa mutant (*Dync1h1^Loa^*) sensory neurons. Here, we describe a new mouse model with a conditional deletion allele of exons 24 and 25 in *Dync1h1*. Homozygous Islet1-Cre-mediated deletion of *Dync1h1* (*Isl1-Dync1h1^−/−^*), which deletes protein from the motor and sensory neurons, is embryonic lethal, but heterozygous animals (*Isl1-Dync1h1^+/−^*) survive to adulthood with ∼50% dynein expression in targeted cells. *Isl1-Dync1h1^+/−^* sensory neurons reveal accelerated growth, as previously reported in *Dync1h1^Loa^* neurons. Moreover, *Isl1-Dync1h1^+/−^* mice show mild impairments in gait, proprioception and tactile sensation, similar to what is seen in *Dync1h1^Loa^* mice, confirming that specific aspects of the Loa phenotype are due to reduced dynein levels. *Isl1-Dync1h1^+/−^* mice also show delayed recovery from peripheral nerve injury, likely due to reduced injury signal delivery from axonal lesion sites. Thus, conditional deletion of *Dync1h1* exons 24 and 25 enables targeted studies of the role of dynein in neuronal growth.

## Teaser

**Summary:** A new mouse model with a conditional deletion of dynein heavy chain 1 confirms that dynein has roles in axonal growth regulation, including axon length sensing, retrograde injury signaling and nerve regeneration.

## INTRODUCTION

Neurons must extend axons over long distances during development to reach their targets and establish functional circuits, and these extended neuronal arbors constitute a vulnerability that is prone to neurological disease (Albus et al., 2013; Sleigh et al., 2019; Terenzio et al., 2017). A number of studies have described neurological disorders resulting from aberrations in signaling pathways implicated in neuronal size control (Nikolaeva et al., 2017; Sundberg and Sahin, 2020) or in intracellular transport (Sleigh et al., 2019). *Dync1h1* encodes the heavy chain of the cytoplasmic dynein 1 motor protein, which plays a key role in retrograde axonal transport in neurons. Various mutations in human *DYNC1H1* have been associated with neurological diseases, including spinal muscular atrophy, Charcot–Marie–Tooth disease, and infantile developmental and epileptic encephalopathy (Hoang et al., 2017; Marzo et al., 2019; Su et al., 2022). Accordingly, a number of dynein mutant mouse lines have been generated as models of neurological disease (Terenzio et al., 2018a).

Prolonged Dync1h1 depletion has been shown to disturb long microtubule transport and alignment in distal axonal segments of superior cervical ganglia neurons, leading to a significant decrease in axonal length (Ahmad et al., 2006). In contrast, we previously demonstrated that partial *Dync1h1* deletion in dorsal root ganglia (DRG) neurons results in axonal growth acceleration (Rishal et al., 2012). The *Dync1h1* legs at odd angles (*Loa*) mouse mutant was originally described with an autosomal dominant mild neurodegenerative disease phenotype (Hafezparast et al., 2003). We subsequently found that heterozygous *Loa* sensory neurons have accelerated axonal growth (Rishal et al., 2012). Thus, the dynein complex links intracellular transport with size control in neurons (Rishal and Fainzilber, 2019; Rishal et al., 2012). However, interpretation of those results was complicated by the fact that the *Loa* mutation is a single nucleotide change rather than a clear loss of function allele. *Dync1h1^Loa^* mice and other described mutants are invariably non-viable as homozygotes, and the adult heterozygous animals have mild to severe neurodegeneration phenotypes (Terenzio et al., 2018a), raising the possibility that specific aspects of the phenotype might be due to effects of the point mutation rather than reduction in dynein levels. An allele deletion model allowing focus on specific neuronal cell types would be of great utility to resolve such issues.

Here, we describe a new conditional deletion allele for *Dync1h1* in the mouse. Islet1-Cre-induced homozygous deletion mice are not viable, but heterozygosity is well tolerated. The heterozygous mice have normal life span and fertility and mutant sensory neurons show the expected decrease in dynein levels, and increased axonal growth. Moreover, Islet1-Cre *Dync1h1^+/−^* adult animals present deficits in motor and proprioception coordination, and delays in recovery from peripheral nerve injury. Thus, this new conditional *Dync1h1* allele enables targeted confirmation of dynein roles in neuronal growth control, and will facilitate comprehensive studies of the physiological roles of dynein.

## RESULTS

### Generation of a conditional deletion allele for *Dync1h1*

We established a floxed *Dync1h1* allele targeting exons 24 and 25 of the *Dync1h1* gene, using available embryonic stem cell (ESC) clones (Skarnes et al., 2011). The floxed allele was generated by transfection of the parental ESC line with an Flp recombinase plasmid, leading to removal of the LacZ-NeoR cassette, and subsequent efficient germline transmission of the *Dync1h1* floxed allele. After recombination, exons 24 and 25 are deleted leading to a frameshift mutation prior to the motor domain of DYNC1H1 protein. This is the same targeting strategy recently used by Baehr and collaborators for analyses of *Dync1h1* roles in the retina and photoreceptor systems (Dahl et al., 2021a,b). Details of the mouse generation strategy and genotyping are presented in Fig. S1 and Materials and Methods.

*Dync1h1* conditional exons 24–25 mice were bred with Islet1 (Isl1) Cre mice. The *Isl1* promoter drives expression in motor and sensory neurons (Srinivas et al., 2001), but also in heart and limb progenitors (Yang et al., 2006). Homozygous *Isl1-Cre* deletions of *Dync1h1* were embryonic lethal, but heterozygous mice (henceforth termed *Isl1-Dync1h1*^+/−^) were viable and used for further characterization. *Isl1-Dync1h1*^+/−^ survived to adulthood and no evident abnormalities were noticed in their postnatal growth rates or life span compared to their wild-type (WT) counterparts.

### Reduced *Dync1h1* expression in heterozygous *Isl1-Dync1h1* mice

**Fig. 1.**
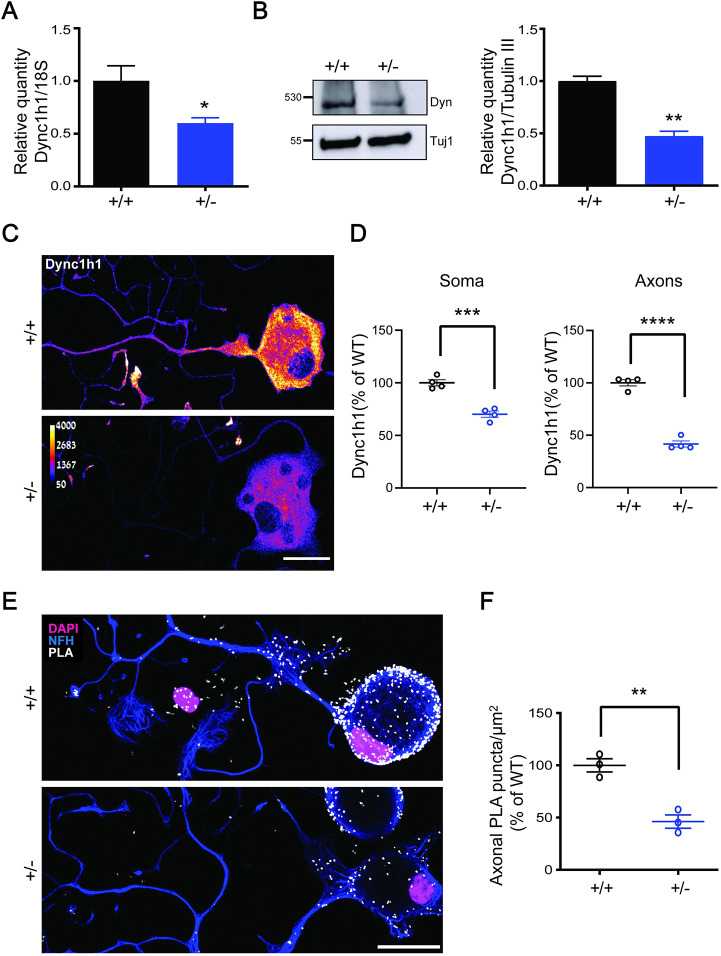
***Dync1h1* mRNA and protein levels are reduced in *Isl1-Dync1h1^+/−^* mice.** (A) qPCR quantification of *Dync1h1* transcript levels using the ΔΔCt method on WT and heterozygous neurons 24 h in culture. Results are mean±s.e.m. (*N*=4 biological repeats). *P<0.05 (unpaired two-tailed *t*-test). (B) Western blot analysis of lysates from WT and heterozygous 24 h neuron cultures. Dyn, Dync1h1; Tubulin III, Tuj1, served as a loading control. Results are mean±s.e.m. (*N*=3 biological repeats). **P<0.01 (unpaired two-tailed *t*-test). (C) Representative images of immunostaining for DYNC1H1 on WT and heterozygous neurons 24 h in culture. Scale bar: 20 μm. (D) Quantification of axonal and somatic immunoreactivity shows significant reduction of DYNH1C1 in mutant mice. Results are mean±s.e.m. (*N*=4 biological repeats). ***P<0.001, ****P<0.0001 (unpaired two-tailed *t*-test). (E) Proximity ligation assay (PLA) for DYNC1H1 and importin β1 in WT and heterozygous neurons fixed after 24 h in culture. Scale bar: 20 μm. (F) Quantification of the PLA shows reduction of axonal signal in *Dync1h1* mutant neurons Results are mean±s.e.m. (*N*=3 biological repeats) **P<0.01 (unpaired two-tailed *t*-test).

We examined dynein mRNA and protein levels in cultured DRG sensory neurons, as *Dync1h1^Loa^* neuron outgrowth investigations were previously conducted in this cell type. Quantitative (q)PCR and western blot analyses revealed a significant reduction of both *Dync1h1* mRNA (Fig. 1A) and protein (Fig. 1B) in neurons cultured for 24 h *in vitro*. This was further confirmed by immunostaining (Fig. 1C,D; Fig. S2A,B). Proximity ligation assay (PLA) was then used to detect spatial coincidence of DYNC1H1 with importin β1, a known component of dynein complexes in axoplasm and cytoplasm (Hanz et al., 2003; Perry et al., 2012). A clear decrease of axonal PLA signals for DYNC1H1–importin β1 in heterozygous neurons confirms that reduced dynein expression is reflected in the prevalence of functional dynein complexes in the mutant (Fig. 1E,F; Fig. S2C,D).

### Neuronal growth phenotype in heterozygous *Isl1-Dync1h1* conditional knockout neurons

**Fig. 2.**
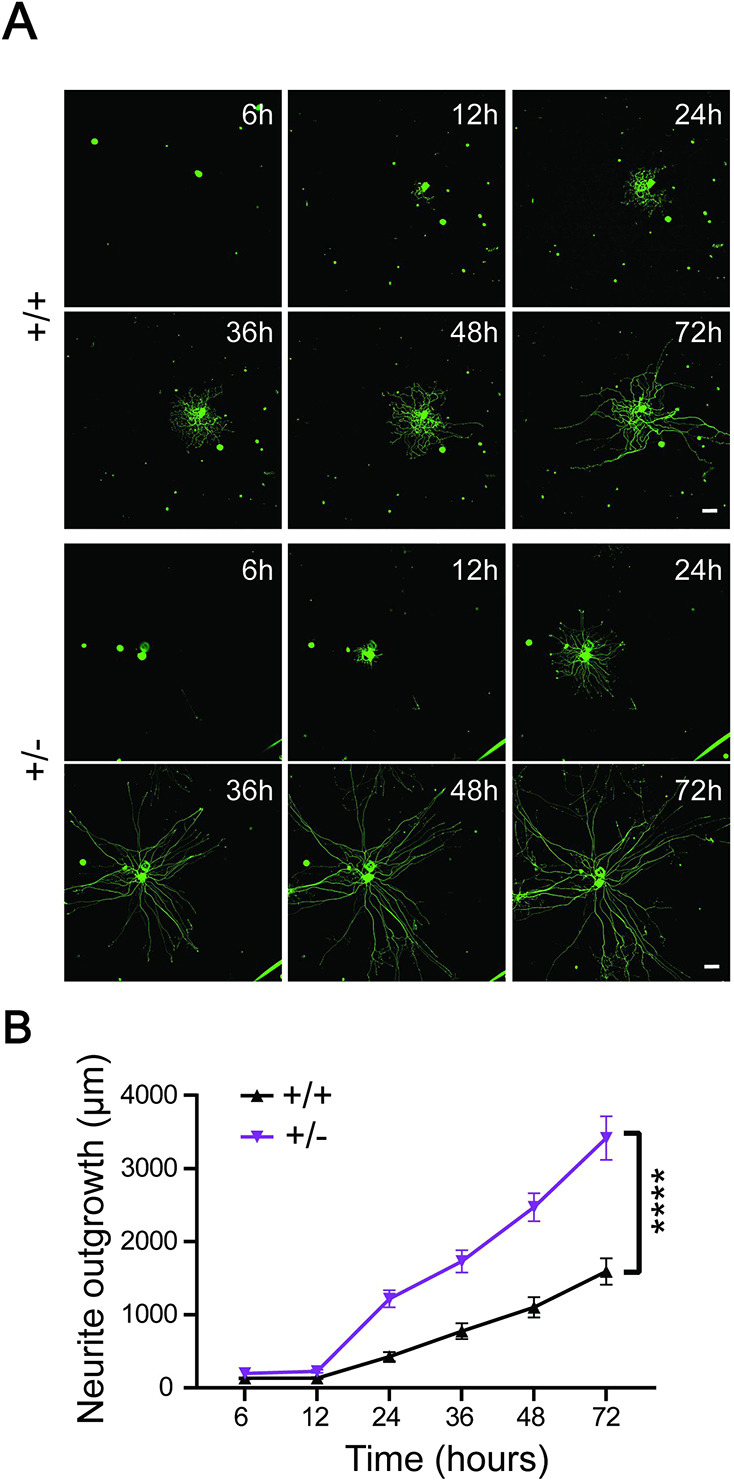
**Time-lapse imaging reveals accelerated growth of *Isl1-Dync1h1^+/−^* sensory neurons.** DRG neurons were plated at a density of ∼25 YFP-expressing cells per well in 96-well plates and outgrowth was monitored by live-cell imaging over 72 h. (A) Representative images of WT and *Isl1-Dync1h1^+/−^* neurons at the indicated time points in culture. (B) Quantification of neurite outgrowth, including only actively growing neurons in the analysis. The experiment was repeated four times. Results are mean±s.e.m. (*n*>315 growing neurons from four biological repeats, ****P<0.0001, two-way ANOVA). Scale bars: 100 µm.

*Isl1-Dync1h1*^+/−^ mice presented abnormal hind limb posture when suspended by the tail (Fig. S3), similar to the phenotype previously reported for whole-body mutant *Dync1h1^Loa^* animals (Hafezparast et al., 2003), and indicative of motor and/or proprioceptive phenotypes (Chen et al., 2007). In previous work, we had demonstrated enhanced neuronal growth of *Dync1h1^Loa^* sensory neurons due to perturbation of a postulated dynein-dependent length-sensing mechanism (Rishal et al., 2012). We tested this prediction of increased growth by cross-breeding *Isl1-Dync1h1*^+/−^ mice with *Thy1-YFP* mice (Feng et al., 2000) to obtain animals that express YFP primarily in large proprioceptive sensory neurons. Time-lapse imaging of axon outgrowth from YFP-expressing neurons in culture reveal significantly accelerated growth of *Isl1-Dync1h1*^+/−^ genotype neurons, both at low culture densities that provide superior conditions for enhanced growth (Fig. 2), and at higher densities comparable to the conditions used in previous studies on *Dync1h1^Loa^* mice (Fig. S4).

### *Isl1-Dync1h1*^+/−^ mice reveal deficits in motor coordination, gait and tactile sensitivity

**Fig. 3.**
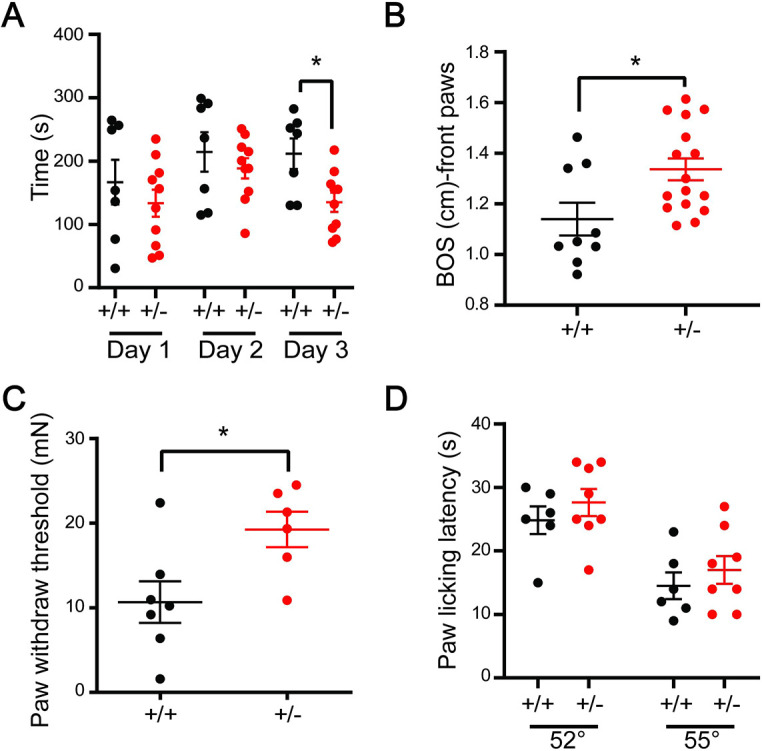
**Motor coordination, gait and tactile sensitivity are impaired in *Isl1-Dync1h1^+/−^* mice, without deficits in heat sensitivity.** (A) Rotarod test. Mice underwent 3 days of training and were subjected to 4 trials each day. The rotarod was accelerated from 0 to 40 rpm in 4 min for the first 2 days and from 0 to 40 rpm in 2 min on the third day. Latency to fall was registered and the average of trials of each day shows significantly reduced performance of the heterozygous mice during the third day. Results are mean±s.e.m. (*n*>7). (B) Walking gait was assessed by catwalk analysis, revealing wider base of support (BOS) in the mutant mice. Results are mean±s.e.m. (*n*>9). (C) Von Frey tests showing reduced mechanical sensitivity of *Isl1-Dync1h1*^+/−^ animals. Results are mean±s.e.m. (*n*>6). (D) Hot plate tests at 52°C or 55°C show no differences in heat sensitivity. Results are mean±s.e.m. (*n*>6). *P<0.05 (unpaired two-tailed *t*-test).

*Dync1h1^Loa^* mice and other dynein heavy chain heterozygous point mutant animals have clear behavioral deficits, including abnormal hind limb positioning and decreased grip strength (Terenzio et al., 2018a). The phenotype was associated with motor and sensory abnormalities, a loss of 50% motor neurons in embryonic day (E)18.5 embryos, and an 86% reduction in muscle spindles in hind limbs by 13 weeks postnatally (Hafezparast et al., 2003). The loss of proprioceptive sensory neurons at the L4 level has been shown to precede the loss of motor neurons, leading to early-onset sensory neuropathy (Chen et al., 2007). We evaluated locomotion deficits, impaired balance and muscular weakness in *Isl1-Dync1h1^+/−^* animals, using catwalk and rotarod tests (Dumont, 2011). *Isl1-Dync1h1^+/−^* mice revealed mild but significant impairments in both assays (Fig. 3A,B). Alterations in tactile sensitivity were also observed (Fig. 3C), whereas, in contrast there were no deficits in heat sensitivity (Fig. 3D). The similar phenotypes of *Isl1-Dync1h1^+/−^* and *Dync1h1^Loa^* animals might be caused by the same motor and sensory abnormalities that have been found in *Dync1h1^Loa^* mice and other dynein heavy chain heterozygous point mutant animals, indicating that these are likely a consequence of partial loss of *Dync1h1* function.

### Impairment in regeneration response to peripheral nerve injury in *Isl1-Dync1h1*^+/−^ mice

**Fig. 4.**
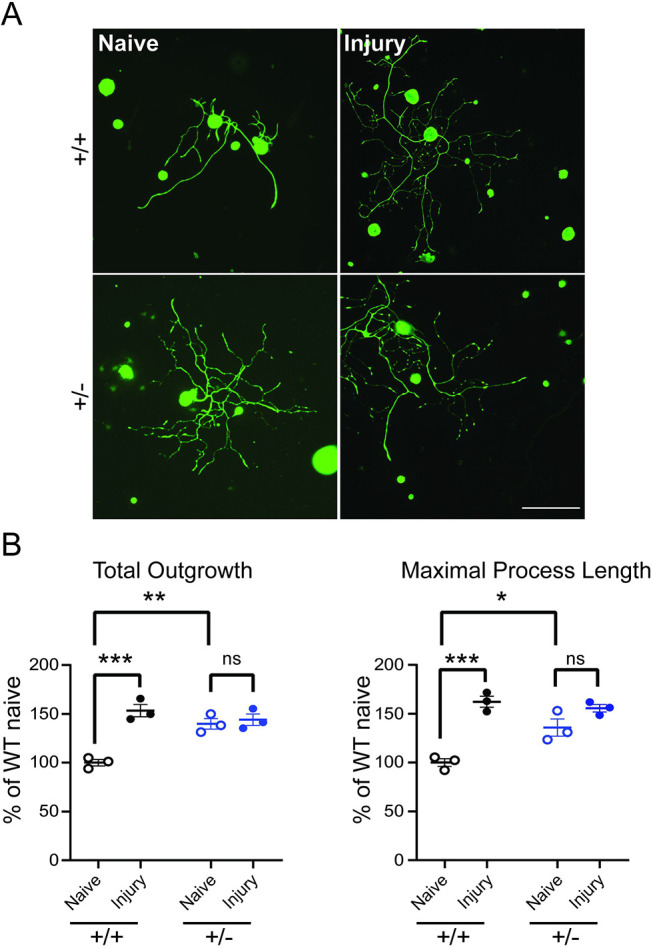
**Attenuated conditioning lesion response in *Dync1h1* heterozygous neurons.** (A) Representative images of sensory neurons isolated from L3, L4 and L5 DRG of WT and heterozygous mice 3 days after unilateral sciatic nerve crush, 24 h in the culture. Scale bar: 200 µm. (B) Quantification of total neurite outgrowth and maximal process length show that *Dync1h1* mutant neurons grow longer than the WT in naive condition, but do not increase their growth further after a conditioning lesion. Data are normalized to the WT naive condition in all graphs. Results are mean±s.e.m. (*N*=3 biological repeats). *P<0.05; **P<0.01; ***P<0.001; ns, not significant (one-way ANOVA).

Previous studies have shown a critical role for dynein in retrograde signaling after peripheral nerve injury (Rishal and Fainzilber, 2014; Terenzio et al., 2017). We therefore examined the injury responses of sensory neurons in *Isl1-Dync1h1^+/−^* mice. Sciatic nerve conditioning lesion (Smith and Skene, 1997) enhances the elongating growth of WT sensory neurons cultured from L3, L4 and L5 DRG, but there was no such effect on *Isl1-Dync1h1^+/−^* neurons (Fig. 4; Fig. S4). The dynein-deficient neurons exhibited enhanced growth in culture under basal conditions, comparable with elongated growth of WT neurons after injury (Fig. 4; Fig. S5). This enhanced growth might mask any potential effect of the conditioning lesion.

**Fig. 5.**
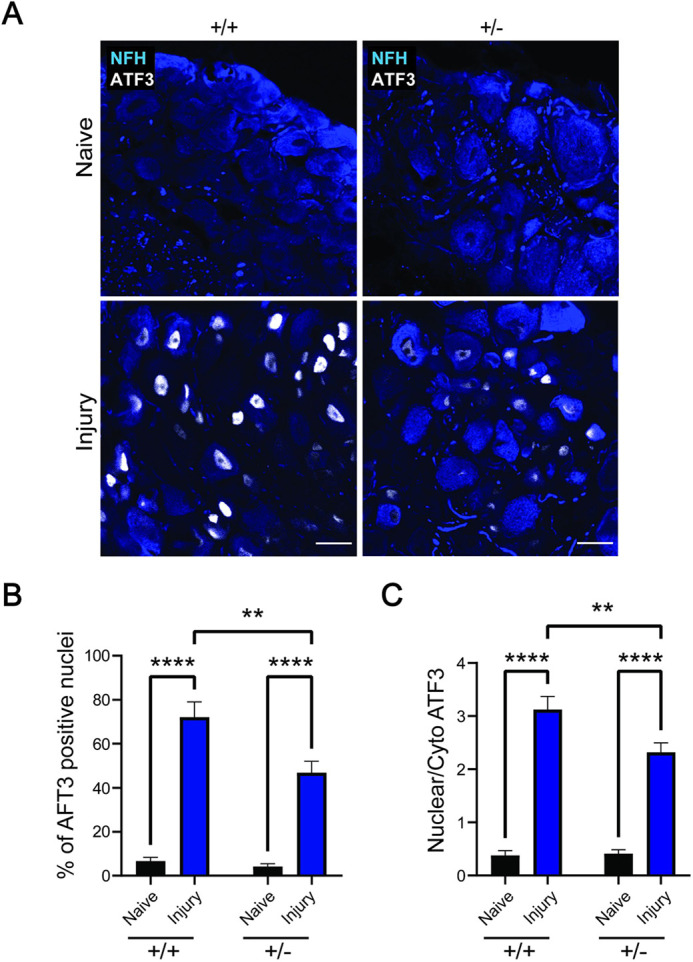
**ATF3 upregulation is significantly attenuated in *Isl1-Dync1h1^+/−^* neurons after injury.** WT and heterozygous mice were subjected to sciatic nerve injury, and L4 DRG were excised 3 days later, processed, sectioned and immunostained as shown. (A) Representative images of WT and heterozygote sections in both naive and injury conditions. ATF3 is clearly upregulated after injury in WT neurons, and to a lesser degree in Dync1h1^+/−^. Scale bars: 30 μm. (B) Percentage of nuclei expressing ATF3 in NFH-positive neurons. Results are mean±s.e.m. (*n*≥8 sections). (C) Quantification of nuclear versus cytoplasmic ATF3. Mean±s.e.m. (*n*≥31 cells) **P<0.01, ****P<0.0001 (one-way ANOVA).

To test whether increased axonal outgrowth of *Isl1-Dync1h1^+/−^* sensory neurons might be due to ‘pre-conditioning’ caused by reduced levels of *Dync1h1*, we assessed expression levels of activating transcription factor 3 (ATF3), a well-established indicator of the regenerative state (Seijffers et al., 2007). Analyses were conducted on DRG from WT or *Isl1-Dync1h1^+/−^* mice, under naive conditions or 3 days after sciatic nerve injury. ATF3 immunoreactivity was low in naive neurons from both WT and *Isl1-Dync1h1^+/−^* mice (Fig. 5A). This finding does not support the possibility of a preconditioning effect due to a decrease in *Dync1h1*. However, *Isl1-Dync1h1^+/−^* neurons show reduced upregulation of ATF3 after injury (Fig. 5), indicative of a deficit in retrograde injury signaling.

**Fig. 6.**
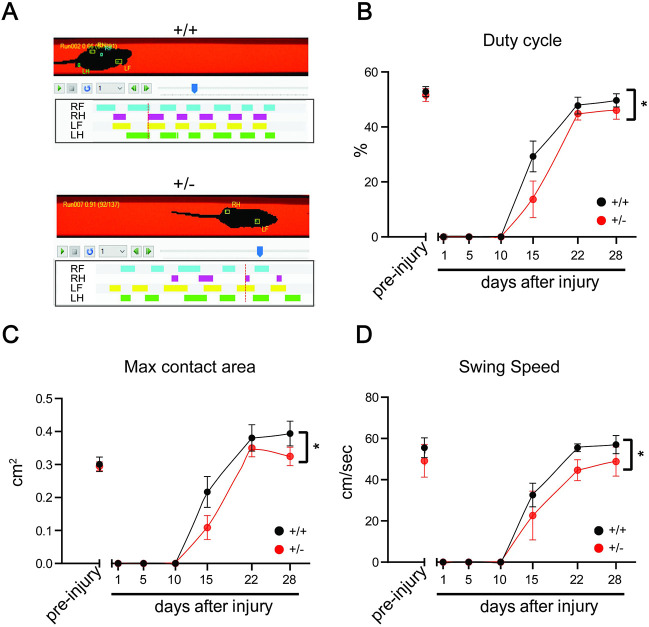
**Gait analysis reveals delayed regeneration of *Isl1-Dync1h1^+/−^* mice.** (A) Representative images of catwalk gait analysis at 15 days after sciatic nerve crush in the right hind (RH) leg. *Isl1-Dync1h1^+/−^* mice show slower recovery of the injured leg. (B–D) Duty cycle, the duration of the contact of the paw with the glass plate, expressed as percentage of a step cycle (B), the maximum contact area, the surface area of the print at the point of maximum contact (C) and swing speed, the speed (cm/sec) of the paw during swing (phase of no contact with the glass plate) (D), were assessed. Results are mean±s.e.m. (*n*≥7). *P<0.05 (two-way ANOVA).

We then proceeded to examine functional recovery from sciatic nerve lesion in *Isl1-Dync1h1^+/−^* mice *in vivo*. Mouse gait parameters were evaluated on a catwalk apparatus before crush injury of the sciatic nerve, and over a time course up to 28 days post-lesion (Fig. 6). As expected, both WT and *Isl1-Dync1h1^+/−^* mice reduced usage of the injured limb immediately after injury, with gradual recovery of different gait parameters over time after lesion (Fig. 6). Differences between WT and *Isl1-Dync1h1^+/−^* mice were apparent through the recovery time course, with *Isl1-Dync1h1^+/−^* mice showing somewhat reduced recovery of the injured limb at the time points assayed during the recovery phase (Fig. 6B–D).

**Fig. 7.**
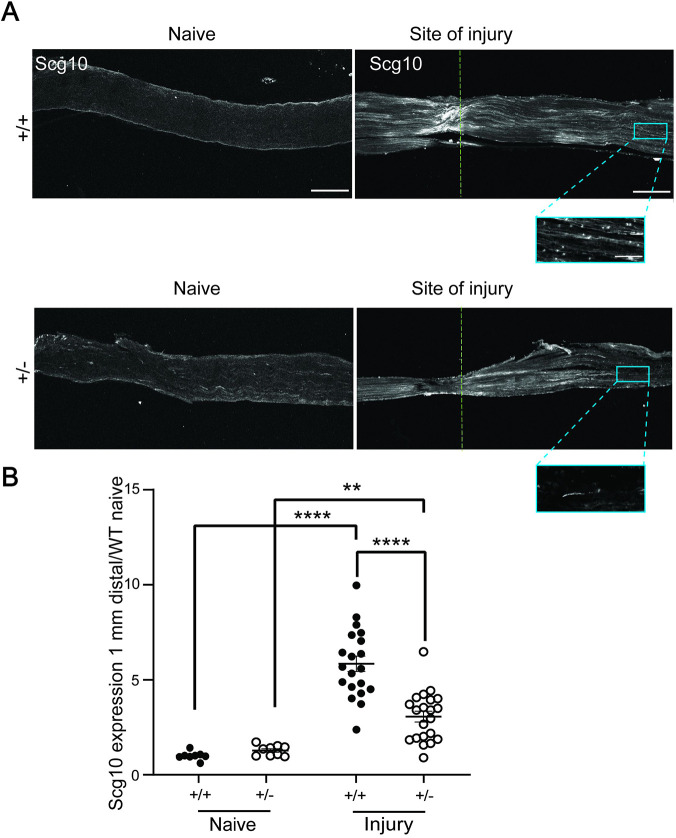
**Delay in regeneration after sciatic nerve injury in *Isl1-Dync1h1^+/−^* mice.** (A) Representative images of longitudinal cryosections of sciatic nerve stained for Scg10 from WT and *Isl1-Dync1h1^+/−^* mice 3 days after injury. Proximal is on the left, distal to the right. Scale bars: 300 μm (main images), 50 μm (higher magnification regions of interest). (B) Quantitative analysis of Scg10 immunoreactivity 1 mm distal to the injury site shows a significant reduction in signal intensity in heterozygous axons compared to WT. Results are mean±s.e.m. (*n*≥8 sections from three biological repeats of the naive, *n*≥20 sections from five biological repeats of injured nerve). **P<0.01, ****P<0.0001, (unpaired two-tailed *t*-test).

To test nerve regeneration after injury, we harvested sciatic nerves from WT and *Isl1-Dync1h1^+/−^* mice 3 days after injury along with naive counterparts, prepared longitudinal cryosections, and immunostained them for Scg10 (also known as Stmn2), a marker for regenerating axons (Shin et al., 2014). There was little or no expression of Scg10 in uninjured axons, and marked upregulation after injury in both genotypes (Fig. 7). However, *Dync1h1^+/−^* axons show significantly lower upregulation of Scg10 than WT axons at 1 mm distal to the injury site (Fig. 7), consistent with the delayed regeneration observed above (Fig. 6).

## DISCUSSION

The dynein heavy chain *Dync1h1* is an essential gene in multicellular organisms, and accordingly all constitutive *Dync1h1* mouse mutants described to date are embryonic lethal when homozygous (Terenzio et al., 2018a). Heterozygous mutants are mostly viable, although they typically have progressively worsening neurodegeneration phenotypes, and given that the mutation is organism-wide, the origin of a specific deficit might be unclear. For example, a number of studies in both *Dync1h1^Loa^* and *Dync1h1^Cra^* mice have suggested that their originally described motor phenotypes might be a secondary consequence of earlier effects in sensory neurons (Chen et al., 2007; Dupuis et al., 2009; Ilieva et al., 2008). Alleles allowing cell type and/or temporal specificity in targeting dynein will be of value in resolving such issues. Moreover, potential confounding or masking effects are less likely in a deletion allele as compared to an expressed mutant with altered amino acid sequence. Indeed, a recent study reported photoreceptor maintenance phenotypes upon conditional deletion of *Dync1h1* exons 24 and 25 in retinal cells (Dahl et al., 2021a) that were not reported previously in *Dync1h1* point mutant mice.

The *Dync1h1* allele described herein enables Cre-driven deletion of dynein with expected effects on dynein expression and function in targeted cell types and tissues. Our previous work had postulated roles for dynein in retrograde injury signaling (Rishal and Fainzilber, 2014), and in intrinsic length-sensing and regulation of axonal growth rates (Rishal et al., 2012). The length-sensing model predicts axon length increase upon partial reduction of dynein levels (Rishal and Fainzilber, 2019; Rishal et al., 2012), and initial experiments in sensory neurons of *Dync1h1^Loa^* mice supported this prediction (Rishal et al., 2012). The results reported above in *Isl1-Dync1h1^+/−^* sensory neurons further support the model, with clearly enhanced outgrowth of heterozygous dynein-knockout neurons. Thus, the *Isl1-Dync1h1^+/−^* model phenocopies the effects of *Dync1h1^Loa^* on sensory neuron growth *in vitro* and on limb proprioception *in vivo*. These functional phenotypes likely reflect similar motor and sensory abnormalities, as previously described in *Dync1h1^Loa^* mice and other dynein heavy chain heterozygous point mutant animals. Taken together, these findings suggest that the sensory and motor phenotypes observed in the *Dync1h1^Loa^* mutant are caused by loss of dynein function, rather than a dominant-negative effect of mutant *Loa Dync1h1* (Garrett et al., 2014; Ori-McKenney et al., 2010).

*In vivo* characterization of *Isl1-Dync1h1^+/−^* mice reveal further parallels with *Dync1h1^Loa^* in limb coordination (Hafezparast et al., 2003), and altered proprioception and gait (Ilieva et al., 2008). The *Isl1-Dync1h1^+/−^* mice also show changed tactile, but not thermal, sensitivity, indicating differential physiological impacts of dynein reduction in distinct sensory neuron subtypes. As regards injury response, *Isl1-Dync1h1^+/−^* neurons do not exhibit enhanced growth after conditioning lesion, and injury-induced upregulation of ATF3 is attenuated in heterozygous neurons. Impaired regeneration was also observed in the *in vivo* analyses, including delayed recovery from sciatic nerve injury, based on gait analysis and immunostaining for the regeneration marker Scg10. Taken together, these data indicate that the enhanced basal neuronal growth of dynein heterozygote neurons is not sufficient to overcome other deficiencies caused by the deletion. Similar findings were described previously for importin β1 (KPNB1) 3′UTR mutant mice, which have deficiencies in retrograde injury signaling and sciatic nerve regeneration (Perry et al., 2012), and also accelerated growth of sensory neurons in culture (Doron-Mandel et al., 2021; Perry et al., 2016). Additional studies utilizing neuron-subtype-specific *Cre* drivers will be required to dissect the underlying mechanisms.

A number of rare neurological diseases have been genetically associated with mutations in the *DYNC1H1* gene. These diseases include motor neuron diseases such as spinal muscular atrophy with lower extremity predominance (SMA-LED) or Charcot–Marie–Tooth disease (Becker et al., 2020; Harms et al., 2012; Scoto et al., 2015; Weedon et al., 2011), and also neurodevelopmental defects (Poirier et al., 2013) and/or epilepsy (Lin et al., 2017; Matsumoto et al., 2021). A retrospective analysis of all published mutations revealed domain-specific genotype-phenotype correlations [i.e. mutations in the dimerization domain with reductions in lower limb strength in DYNC1H1-NMD (neuromuscular disorders) and motor domain with cerebral malformations in DYNC1H1-NDD (neurodevelopmental disorders) (Becker et al., 2020)]. Elucidating the origin of these diverse clinical presentations will benefit from the availability of the dynein deletion allele model, potentially allowing determination of specific cell types linked to each disease.

To summarize, our results on heterozygous *Dync1h1* deletion in sensory neurons confirm model predictions and previous findings from *Dync1h1^Loa^* mice on a dynein-dependent mechanism that regulates neuronal growth. The *Dync1h1* conditional allele provides a new model for targeted studies on dynein functions in specific cell types.

## MATERIALS AND METHODS

### Animals

All animal experiments were approved by the IACUC (Animal Care and Use Committee) at the Weizmann Institute of Science. Mice were bred and maintained at the Veterinary Resources Department. Experiments were carried out on animals between 2 and 6 months old. In this work, all mice used for experimentation have a *BALB/cN/C57BL/6/129×1/SvJ/ICR* mixed genetic background strain. The *C57BL/6YFP16* mice (Feng et al., 2000) and *Isl1-Cre (129X1/SvJ/ICR)* (Srinivas et al., 2001) mice were maintained at the Veterinary Resources of the Weizmann Institute. According to the IACUC guidelines, animals were kept at 24.0±0.5°C in a humidity-controlled room under a 12 h light–12 h dark cycle with all the time access to food and water. Mice used for behavioral tests were kept at the same conditions in a reverse dark–light cycle room.

### Mutant mouse line establishment

The *Dync1h1* conditional mutant mouse line was established at the Institut Clinique de la Souris - PHENOMIN (http://www.phenomin.fr). Three clones EPD0284-5-C04, EPD0284-5-D04 and EPD0284-5-H02 were ordered from the Mutant Mouse Resource Research center (https://www.mmrrc.org/). All three clones contained the standard knockout first with the potential conditional International Mouse Phenotyping Consortium (IMPC) allele. The critical exons 24 (ENSMUSE00000116212) and 25 (ENSMUSE00000116247) are floxed. The deletion of these exon leads to a frame shift. Proper homologous recombination was confirmed by Southern blotting using an internal probe (Neo probe) (Codner et al., 2021). Four restriction enzymes were used, all showed a single band at the expected size, confirming the correct recombination at the *Dync1h1* locus for all three clones. Two additional digests were used to confirm the presence of the 3′ LoxP. This latter site was also confirmed by PCR. The three clones were karyotyped by chromosome spreading and Giemsa staining and microinjected in BALB/cN blastocysts. Even if good chimeras were obtained (until 85% of percentage of chimerism), none gave germline transmission.

Hypothesizing that the absence of germline transmission might indicate lethality upon implementation of the knockout first strategy, we decided to remove the flipped LacZ-NeoR cassette directly in an ESC clone *in vitro* in order to obtain the conditional knockout allele (tm1c) (Fig. S1). Electroporation with a plasmid expressing the FlpO recombinase (cloned under a pCAG promoter) was performed on clone EPD0284-5-D04. Different subclones were obtained and analyzed by PCR. Two PCR sets were used and confirmed the excision of the flipped cassette. One positive subclone was microinjected in BALB/cN blastocysts, male chimeras were obtained and germline transmission was achieved (tm1c allele; conditional knock-out).

Genotyping of *Isl1-Dync1h1*^+/−^ mice was performed on genomic DNA isolated from ears as shown in Fig. S1B,C using following primers: LoxP F, 5′-CCATTGTCCCCCTGGTTTCATCC-3′; LoxP R, 5′-CTGTGTCAGTTGCAGACAGTTTCTACG-3′; Intron23 F, 5′-GCTGCCTGAACTCCCAGTTTTCCG-3′.

The presence of Cre gene was correlated with excision of the floxed exons, that is knockout, as shown in Fig. S1B.

### Cultures and antibodies

Culture of sensory neurons from DRG were performed as previously described (Terenzio et al., 2018b). Adult DRG were dissociated by enzymatic treatment (papain and a mix of collagenase/dispase), triturated in Hanks’ balanced salt solution (HBSS)-supplemented with 10 mM glucose and 5 mM HEPES pH 7.35, and recovered by centrifugation (1000 ***g*** for 8 min) through 20% Percoll. Sensory neurons obtained from this procedure were then subjected to immunostaining. Primary antibodies used in this study are the following: chicken anti-NFH (Abcam, ab72996, 1:2000), rabbit anti-Dync1h1 (Proteintech, 12345-1-AP, 1:500), mouse anti-importin β1 (monoclonal, generated in-house), rabbit anti β-III tubulin (Abcam, ab18207, 1:2000), recombinant rabbit monoclonal anti-ATF3 (Abcam, ab207434, 1:1000, for IF) and rabbit polyclonal anti-Stathmin2/Scg10 (Novus, NBP1-49461, 1:1000, for immunofluorescence). Secondary antibodies used for immunostaining are anti-chicken-IgY, -mouse-IgG or -rabbit-IgG conjugated to Alexa Fluor 488, 594 or 647 (Jackson ImmunoResearch, 1:1000). Secondaries for immunoblotting were HRP-conjugated anti-rabbit-IgG antibodies (Bio-Rad Laboratories, 1:10,000).

### *Dync1h1* gene expression analysis by qPCR

The mRNA level of *Dync1h1* was quantified by qPCR analysis. RNA was extracted from DRG neurons grown 24 h in culture using the Oligotex mRNA Mini kit (Quiagen). Superscript III (Invitrogen) was used to synthesize cDNA and qPCR reactions were prepared in a total volume of 20ul using PerfeCTa SYBR Green (Quanta Biosciences, Gaithersburg, USA) and performed on a ViiA7 System (Applied Biosystem). *18S* was used as housekeeping gene for normalization, and data were analyzed using the comparative ΔΔCt method (Livak and Schmittgen, 2001). The sequences of the primers are as follows: 18S forward, 5′-AAACGGCTACCACATCCAAG-3′; 18S reverse, 5′-CCTCCAATGGATCCTCGTTA-3′; Dync1h1 forward, 5′-CCAACAGCTTGGCGTTCAT-3′; and Dync1h1 reverse, 5′-GGGACGACACTGGCTTGTCT-3′.

### DYNC1H1 protein expression analysis by western blotting

Western blot analysis was conducted on DRG neurons cultured for 24 h. Cells were harvested and lysed in RIPA buffer. Proteins were then boiled in 5× Laemmli sample buffer, fractionated by SDS-PAGE, and transferred to 4–15% gradient gels using a Bio-Rad transfer apparatus according to the manufacturer's protocol. Membranes were incubated for 1 h at room temperature in a blocking solution of 5% milk in 10 mM Tris-HCl pH 8.0, 150 mM NaCl and 0.5% Tween 20 (TBST), washed and incubated with the rabbit anti-Dync1h1 antibody and rabbit anti-β-III tubulin antibody overnight at 4°C. The following day, they were washed and incubated with HRP-conjugated anti-rabbit-IgG antibodies for 1 h. Images were captured with the ECL system (Amersham Biosciences) and the signal quantified using Fiji software.

### PLA labelling

Proximity ligation assay (PLA) (Gullberg et al., 2004) was used to detect spatial coincidence of DYNC1H1 and importin β1 proteins. PLA was performed according to manufacturer's instructions using Duolink (Sigma; PLA probe anti-mouse minus DUO92004, anti-rabbit plus DUO92002, and detection kit red DUO92008). After the PLA protocol, cells were immunostained with chicken anti-NFH antibody. The images were taken using Fluoview (FV10i), a fully automated confocal laser–scanning microscope at a 60× magnification with a water immersion objective (Olympus UPLANSAPO, NA 1.2). The PLA signal was quantified with the ‘analyze particles’ function of Fiji software, using a mask based on intensity of NFH staining and dividing the number of PLA-positive puncta by the NFH stained area excluding cell body.

### Axon outgrowth analysis

For outgrowth analysis, *Isl1-Dync1h1^+/−^* and WT mice were crossed with *Thy1–YFP* mice (Feng et al., 2000). DRG neurons from adult mice were plated on a 96-well glass-bottom plate (Cellvis) coated with 0.01% poly-L-lysine (Sigma P4832; 50 ml) and laminin (7 μg/ml final concentration; Thermo Fisher Scientific; 23017015). After seeding, neurons were imaged at 6, 12, 24, 36, 48 and 72 h after plating using an ImageXpress Micro (Molecular Devices), a fully automated spinning disc confocal microscope with a built-in CO_2_ incubator, at a 10× magnification. The cells were analyzed using the MetaXpress software (Molecular Devices). The total outgrowth parameter was defined as the sum of all process lengths. The maximum process length parameter was defined as the sum of the length of the longest process plus the lengths of all branches associated with it. Only growing cells were included in analysis.

### Conditioning lesion

For conditioning lesion experiments, mice were subjected to unilateral sciatic nerve injury and, after 3 days, DRG L3, L4 and L5 were dissociated and cultured for 24 h. The plating was done on a DMEM/F12 medium supplemented with 1× N1 medium supplement (Sigma; N6530) and 10% fetal bovine serum, as was done previously (Hanz et al., 2003). Neurons were then fixed in 4% paraformaldehyde, stained with NFH, and subjected to morphological analysis. Images were captured at 10× magnification on ImageXpress Micro (Molecular Devices), and outgrowth was analyzed using the MetaXpress software (Molecular Devices). Only ‘sprouted’ cells were included in analysis. A cell was considered sprouted when the maximum length of the process was equal to twice the cell diameter.

### Immunohistochemistry on cultured neurons

Sensory neurons from WT and *Isl1-Dync1h1^+/−^* mice were grown on glass coverslips coated with poly-L-lysine and laminin for 24 or 48 h and then fixed using 4% paraformaldehyde (PFA) in 1× PBS. Blocking and permeabilization was done with 10% donkey serum and 0.2% Triton X-100 in PBS for 1 h. Coverslips with neurons were incubated overnight at 4°C with chicken anti-NFH (Abcam, ab72996, 1:2000) and rabbit anti-Dync1h1 (Proteintech, 12345-1-AP, 1:500). The next day, they were washed three times in 1× PBS and incubated for 1 h with donkey anti-chicken-IgY conjugated to Alexa Fluor 488, donkey anti-rabbit-IgG conjugated to Alexa Fluor 647 (1:1000; Jackson ImmunoResearch) and DAPI (Abcam, ab228549, 1:5000). After three washes in 1× PBS, coverslips were rinsed in ddH_2_O and mounted with Fluoromount-G^TM^.

### Immunohistochemistry on cryosections

L4 DRG and sciatic nerves of adult WT and *Isl1-Dync1h1^+/−^* mice were harvested 3 days after crush injury together with non-injured controls. Then, the tissue were fixed in 4% PFA for 12 h at 4°C, washed three times in 1× PBST (10 min each), incubated overnight in 30% sucrose and subsequently embedded in OCT freezing medium (Tissue-Tek O.C.T. Compound, Sakura Cat.#4583). The 15 µm thick sections were cut with a cryostat in set of three and mounted on to glass slides. For the immunostaining, one set of each biological repeat was used. The sections were re-hydrated in 1× PBS, blocked and permeabilized with 10% donkey serum and 0.2% Triton X-100 in 1× PBS for 1 h at room temperature, and then incubated overnight with primary antibodies at 4°C. DRG sections were incubated in chicken anti-NFH (Abcam, ab72996, 1:2000) and rabbit anti-ATF3 (abcam, ab207434, 1:1000), whereas longitudinal sections of nerves were incubated in chicken anti-NFH and rabbit anti-Scg10 (Novus, NBP1-49461, 1:4000). Primary antibody solutions were carefully removed, and slides were washed three times (10 min each) at room temperature with 1× PBS and then incubated with the secondary antibodies donkey anti-chicken-IgY conjugated to Alexa Fluor 594 and donkey anti-rabbit-IgG conjugated to Alexa Fluor 488 (1:1000 each; Jackson ImmunoResearch) and DAPI (Abcam, ab228549, 1:5000) for 1 h at room temperature. After three 5-min washes in 1× PBS, slides were mounted with Fluoromount-G^TM^.

All fluorescence images were captured using an Olympus FV1000 Confocal laser-scanning microscope at 60× magnification with oil-immersion objective (Olympus UPLSAPO, NA 1.35) or using Fluoview (FV10i), a fully automated confocal laser–scanning microscope, with water immersion objective (Olympus UPLANSAPO 60X, NA 1.2). Image analysis was performed using Fiji software (Schindelin et al., 2012) creating a mask based on NFH channel and quantifying the mean intensity of our protein of interest. To test axonal regeneration after SN crush, the Scg10 intensity level at a distance of 1 mm distal to the injury site was divided by the Scg10 intensity level in the naive nerve. For the ATF3 experiment, we quantified both the protein intensity and the percentage of ATF3-positive nuclei of NFH neurons.

### Behavioral profiling

All assays were performed under dim illumination (∼10 lx) during the ‘dark’ active phase of the diurnal cycle. Mice were tested with the ROTOR-ROD, catwalk, von Frey and hot plate tests as follows.

For ROTOR-ROD, mice underwent 3 days of training on the rotarod, accelerating from 0 to 40 rpm in 4 min (inclination of 10 rpm/min) during day 1 and 2. On the third day, the rotarod was accelerated from 0 to 40 in 2 min (20 rpm/min). Each day, mice were subjected to four trials with a 2 min break in between as shown previously (Ezra-Nevo et al., 2018). Latency to fall (s) was recorded and the average of the four trials per day was considered.

The catwalk test was carried on as previously described (Terenzio et al., 2020). Motivation was achieved by placing the home cage at the runway end and each mouse was tested three to five times. The collected data were analyzed using the Catwalk Ethovision XT10.6 software (Noldus Information Technology, The Netherlands). Mice showed differences in the base of support parameter (BOS), defined as the average width (cm) between the paws during each step cycle.

For the Von Frey test, mechanical sensitivity was tested by pressing filaments of different diameters on the plantar surface of the paw of the mouse. Before starting the test, mice were habituated in chambers suspended above the test apparatus wire mesh grid for an hour. Once the mice were calm, the test with the Von Frey filaments started and the response was considered positive if the paw was sharply withdrawn upon filament application, starting with 13.7 millinewton filaments and then progressing in an up-down method, as previously described (Marvaldi et al., 2020).

For the hot plate test, mice were tested for heat sensitivity at temperatures of 52°C and 55°C. Each mouse was placed in a 20 cm high Plexiglas box on the heated metal surface and the latency to initiate a nociceptive response (licking, paws shaking, jumping) was recorded.

For the catwalk assay to test regeneration *in vivo*, we made use of the catwalk test described above. Before sciatic nerve injury, mice were trained on the Catwalk apparatus and a baseline of both genotypes was assessed 1 day before the crush. The locomotion recovery of the mice was observed during the day 1, 5, 10, 15, 22 and 28 post-injury showing reduced recovery of the *Isl1-Dync1h1^+/−^* mice.

### Quantification and statistical analysis

Data shown represent mean±s.e.m., unless otherwise noted. *N* represents number of independent biological repeats, *n* represents number of cells or sections. Statistical analyses and graphs were generated using GraphPad Prism 9 software. Pairwise analyses were conducted with an unpaired two-tailed *t*-test (Figs 1 and 3B,C,D; Fig. S2). Groupwise analyses were conducted by one-way ANOVA for one-factor analyses assuming equal variances (Figs 4 and 5; Fig. S5) or two-way ANOVA where >1 factor was analyzed (Figs 2, 3A and 7; Fig. S4). Statistically significant *P*-values are shown as *P<0.05, **P<0.01, ***P<0.001 and ****P<0.0001.

## Supplementary Material

10.1242/joces.260220_sup1 Supplementary information
