# Supplementary material for: A conditional null allele of Dync1h1 enables targeted analyses of dynein roles in neuronal length sensing
Source: J Cell Sci. 2022 Oct 31;136(5):jcs260220. doi: 10.1242/jcs.260220 (PMC9687604; doi:10.1242/jcs.260220)
Supplement: Supplementary information [file joces-136-260220-s1.pdf]

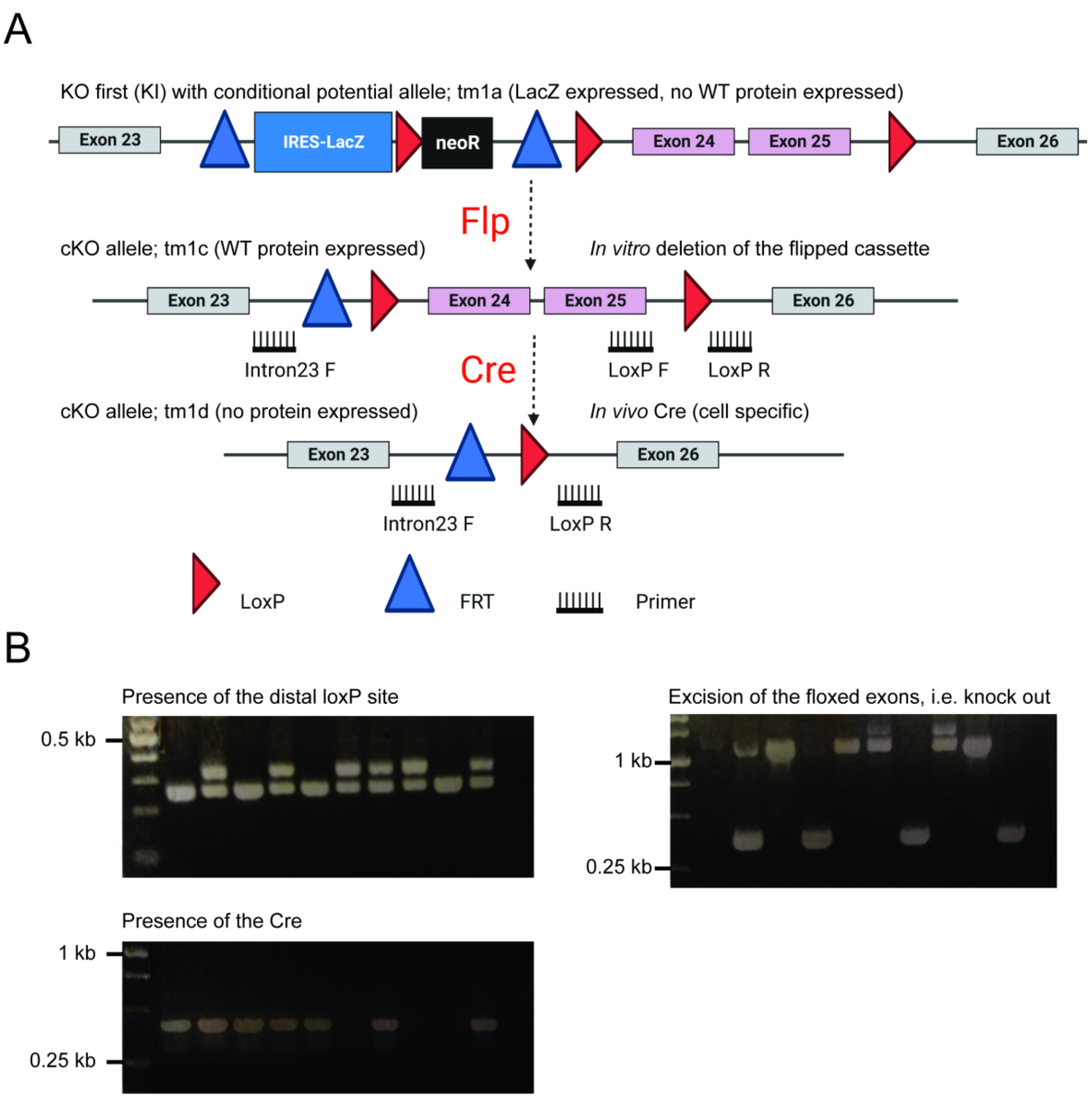

C

PCR fragments expected size (bp):

Region analyzed	Primers used	cKO allele tm1c	KO allele tm1d	WildType allele (WT)
Presence of the distal loxP	loxPF/loxPR	316	---	249
Excision of the floxed exon(s), i.e. knock out	Intron23F/loxPR	1435	372	1164

Fig. S1. Generation of the mutant mouse line. A, Conditional *Dync1h1* allele with FRT sites located around IRES-LacZ and NeoR cassette allowing Flp recombination resulted in IRES-LacZ-NeoR cassette deletion and obtaining conditional allele with WT

Journal of Cell Science • Supplementary information

protein expression. loxP sites of the floxed *Dync1h1* allele are located in introns 23 and 25. Following Cre-induced recombination, the null allele loses exons 24 and 25 resulting in *Dync1h1* KO. **B,** Validation of the presence of the 3' loxP site by PCR reaction in conditional allele before Cre-induced recombination in other tissue where Cre is not expressing, used in genotyping from tails or ears, left upper panel. Right upper panel represents Cre-induced recombination with exons deletion that takes place only in the present of Cre, left lower panel. **C,** Sequences and location of primers used for genotyping.

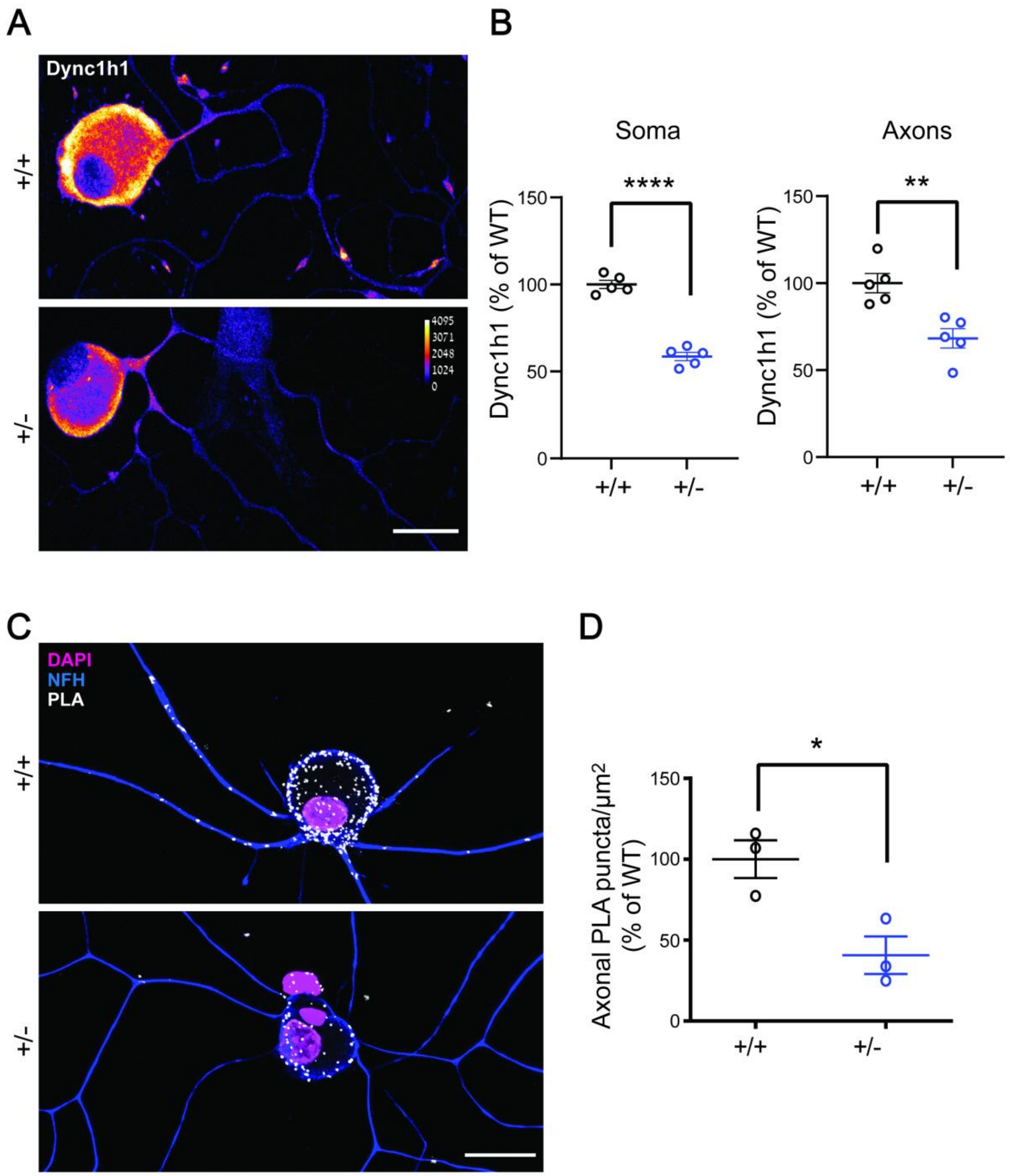

Fig. S2. Dync1h1 protein levels are reduced in *Isl1-Dync1h1*$^{+/-}$ mice 48 hr in culture. A, DYNC1H1 immunostaining on wild-type and heterozygous neurons 48 hr in culture, scale bar 20 µm. **B,** Quantification of (A), Mean ± SEM, N=5 biological repeats, **, **** p< 0.01, 0.0001, respectively, unpaired t-test. **C,** PLA for DYNC1H1 and Importin β1, Scale bar 20 µm. **D,** PLA quantification, Mean ± SEM, (N=3 biological repeats, * p< 0.05, unpaired t-test).

Journal of Cell Science • Supplementary information

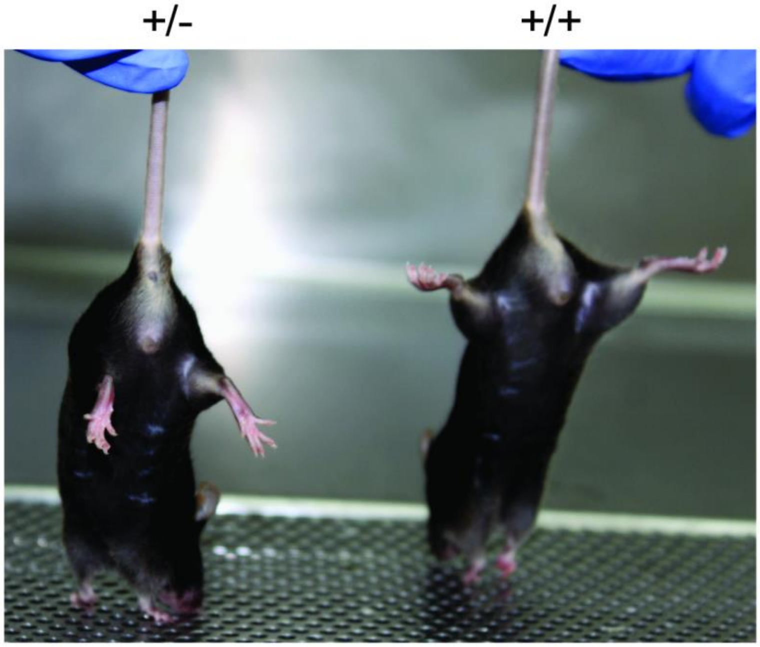

Fig. S3. *Isl1-Dync1h1*$^{+/-}$ mice present an abnormal clenched hind limb posture when suspended by the tail, resembling the characteristic "legs at odd angles" phenotype of *Dync1h1*^{Loa} mice.

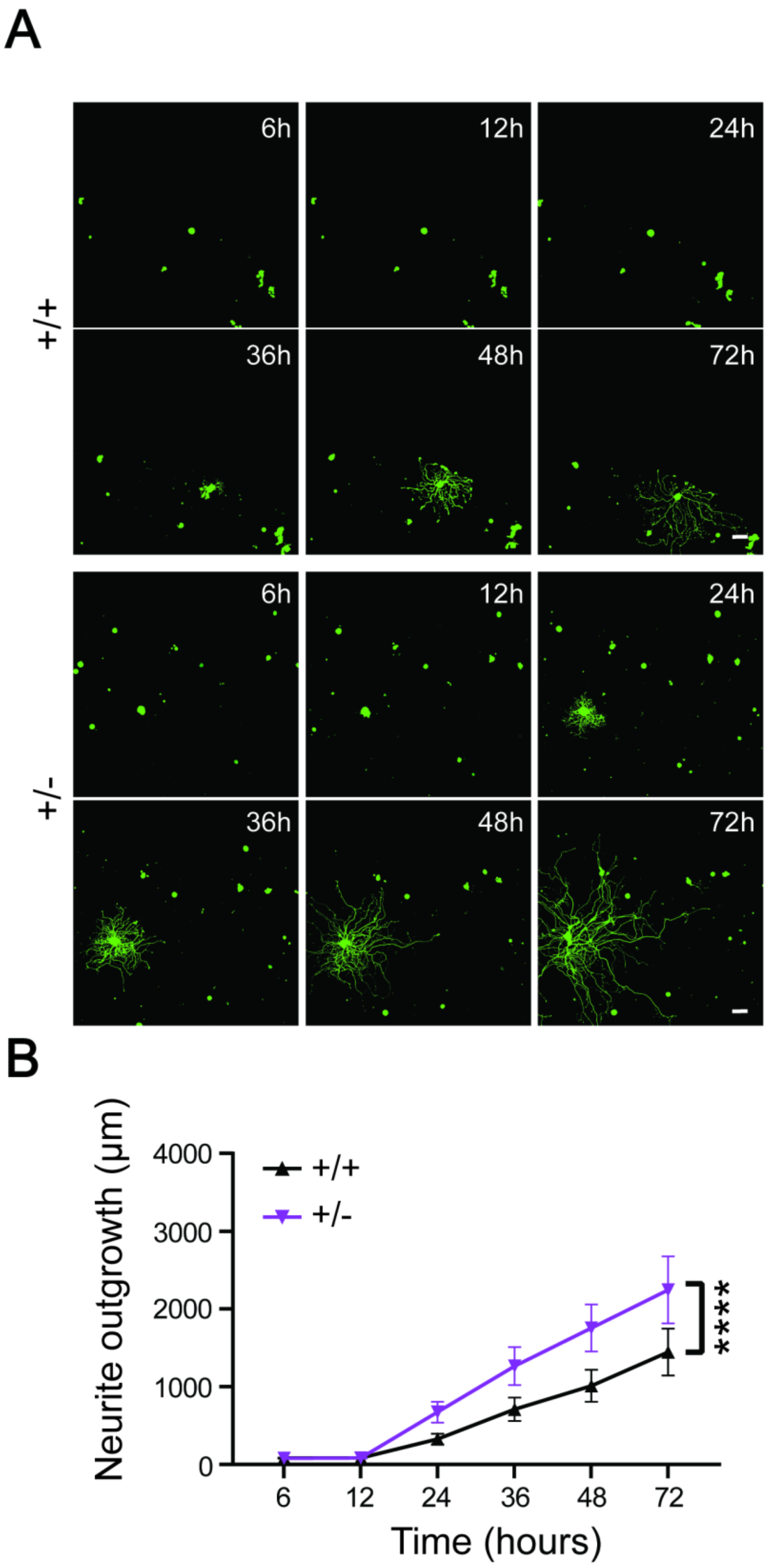

Journal of Cell Science • Supplementary information

Fig. S4. Time-lapse imaging reveals accelerated growth of *Isl1-Dync1h1*$^{+/-}$ sensory neurons also when plated at higher density. Experimental conditions as inFigure 2, except for plating density which was 45 YFP-expressing neurons per well in 96-well format. **A,** Representative images of wild type and *Isl1-Dync1h1*$^{+/-}$ neurons at the indicated time points in culture. **B,** Quantification of neurite outgrowth, including only actively growing neurons in the analysis. Mean ± SEM (n> 128 growing neurons, **** p<0.0001, two way ANOVA). Scale bars 100 µm.

Journal of Cell Science • Supplementary information

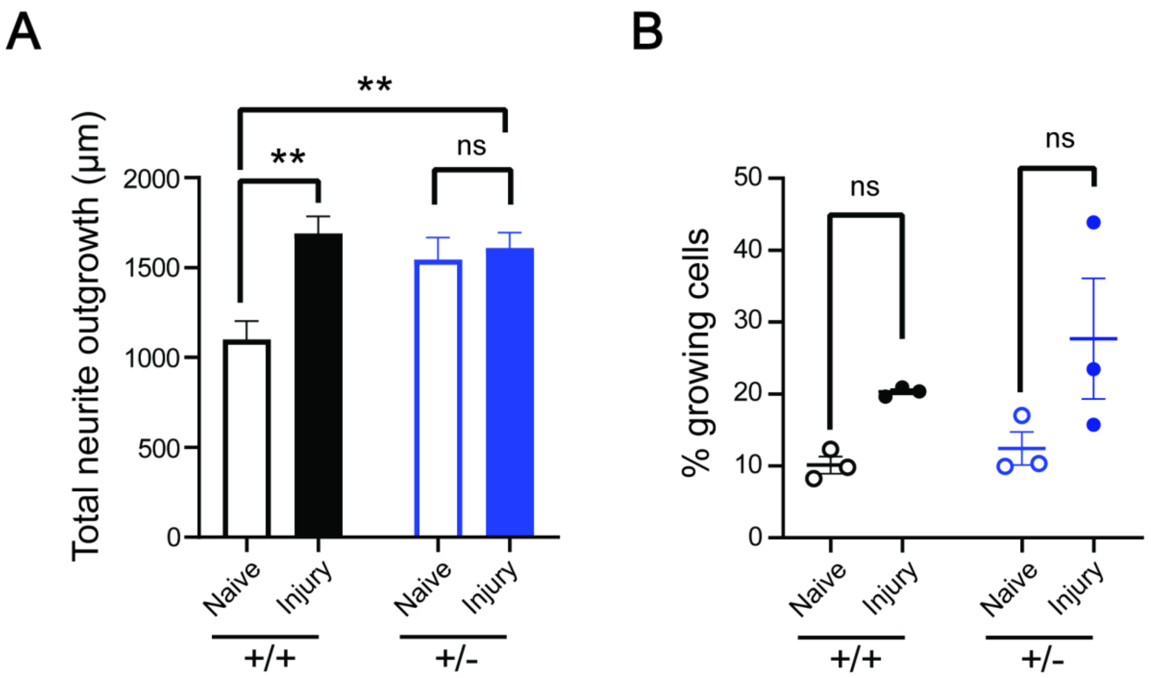

Fig. S5. *Dync1h1+/-* neurons are not affected by conditioning lesion. A, Analysis of neurite outgrowth was performed on the total number of cells in 3 biological repeats and including only actively growing neurons. Mean ± SEM (n > 199 growing neurons, ** p<0.01, one way ANOVA). **B,** Quantification of percentage of growing cells. In either wild type or heterozygous condition, the number of growing cells is not statistically different after injury. Mean ± SEM, (N=3 biological repeats, one way ANOVA).

Journal of Cell Science • Supplementary information
