# Supplementary material for: A conditional null allele of Dync1h1 enables targeted analyses of dynein roles in neuronal length sensing
Source: J Cell Sci. 2022 Oct 31;136(5):jcs260220. doi: 10.1242/jcs.260220 (PMC9687604; doi:10.1242/jcs.260220)
Supplement: Supplementary file 1 [file joces-136-260220_review_history.pdf]

Journal of Cell Science

A conditional null allele of *Dync1h1* enables targeted analyses of dynein roles in neuronal length sensing

Agostina Di Pizio, Letizia Marvaldi, Marie-Christine Birling, Nataliya Okladnikov, Luc Dupuis, Mike Fainzilber and Ida Rishal
DOI: 10.1242/jcs.260220

Editor: Anne Straube

Review timeline
Original submission: 9 May 2022
Editorial decision: 15 June 2022
First revision received: 15 September 2022
Accepted: 3 October 2022

Original submission

First decision letter

MS ID#: JOCES/2022/260220

MS TITLE: A conditional null allele of Dync1h1 enables targeted analyses of dynein roles in neuronal length sensing

AUTHORS: Agostina Di Pizio, Letizia Marvaldi, Marie-Christine Birling, Nataliya Okladnikov, Luc Dupuis, Mike Fainzilber, and Ida Rishal
ARTICLE TYPE: Research Article

We have now reached a decision on the above manuscript.

To see the reviewers' reports and a copy of this decision letter, please go to: https://submit-jcs.biologists.org and click on the 'Manuscripts with Decisions' queue in the Author Area. (Corresponding author only has access to reviews.)

As you will see, the reviewers raise a number of substantial criticisms that prevent me from accepting the paper at this stage. They suggest, however, that a revised version might prove acceptable, if you can address their concerns. If you think that you can deal satisfactorily with the criticisms on revision, I would be pleased to see a revised manuscript. We would then return it to the reviewers.

Please ensure that you clearly highlight all changes made in the revised manuscript. Please avoid using 'Tracked changes' in Word files as these are lost in PDF conversion.

I should be grateful if you would also provide a point-by-point response detailing how you have dealt with the points raised by the reviewers in the 'Response to Reviewers' box. Please attend to all of the reviewers' comments. If you do not agree with any of their criticisms or suggestions please explain clearly why this is so.

Reviewer 1

Advance summary and potential significance to field

© 2022. Published by The Company of Biologists under the terms of the Creative Commons Attribution License (https://creativecommons.org/licenses/by/4.0/).

The authors have created a mouse that expresses about half as much dynein in certain cell/neuron population. This was done by deleting a couple of exons. It's not 100% convincing that the end result is as simply interpreted as half as much dynein, but the authors are sufficiently transparent about what they did that this can be debated as their data are discussed by readers. In that sense, I think it is okay. In fact it's potentially a useful tool for the future in that some of the animal's symptoms are reminiscent of human diseases such as those with gait defects.

Overall, the authors push their interpretation that the phenotype is due to a role they posited in the past for dynein in regulating the size of cells. It's a bit baffling in the sense that other published studies suggest stunted growth with dynein inhibition, not enhanced growth, but I suppose that can be interpreted as more substantial levels of inhibition than what the author's report here.

Mechanistically, the paper does very little on the cell biological consequences of less dynein. The manuscript is very short and in some ways seems like a sliver of what a paper should be with the generation of this new mouse.

Comments for the author

The manuscript is sufficiently interesting and the new mouse sufficiently important that I am okay with this paper for JCS being short. I do think the authors should do a much better job with the expansive literature on how dynein contributes to axon growth, health and vitality. It seems a bit odd to interpret everything in such narrow terms as they do.

<u>Reviewer 2</u>

Advance summary and potential significance to field

In this manuscript, Di Pizio et al. describe a new mouse model allowing deletion of Dync1h1 in a targeted cell type, which enables targeted studies of the role of dynein in neuronal growth. The authors show that Isl1-cre mediated deletion of dynein heavy chain (Isl1-Dync1h1+/-) in sensory neurons leads to an accelerated growth phenotype along with mild impairments in gait, proprioception and tactile sensation and slightly impaired recovery from peripheral nerve injury. This represents the characterization of very useful model for the field, which will facilitate comprehensive studies of the roles of dynein in a cell specific manner.

Comments for the author

There are some points that need to be revised to facilitate the logical flow and strengthen some of the conclusions. As presented, it is difficult to reconcile the effect on in vitro growth and in vivo phenotypes. Consider revising the text in abstract and throughout the whole manuscript to better correlate these two important findings if they are indeed correlated, or state clearly that these are two independent phenotypes observed in the mutant mouse.

Major points

1. The authors need to better explain why Isl1-Dync1h1+/- neurons exhibit enhanced growth in culture but at the same time show impaired recovery after nerve injury. It is also not clear how the in vitro accelerated growth relates to the in vivo phenotypes that include mild impairments in gait, proprioception and tactile sensation. As presented, one expects these two observations to be causally related. If this is not the case, it would be important to revise the text to present these two observations as independent findings that highlight the multiple function of dynein. If there is a causal relation, then innervation of muscle or skin targets could be used to see if increased growth in culture correlates with altered or impaired innervation in vivo.
2. The interpretation of the results in Figure 4 is that conditioning lesion does not enhance growth in Isl1-Dync1h1+/- neurons. The author discuss that this is because growth is already enhanced in naïve condition. Is that solely due to the length sensing mechanism previously proposed, or to the fact that the neurons are conditioned already by reduced levels of dynein heavy chain? A staining for ATF3, as a proxy for the regenerative state, in DRG sections could help address this point and determine if sensory neurons are in an active growth mode at baseline. Furthermore, analysis of nerve regeneration in sciatic nerve in vivo 1 or 3 days post injury could help understand if the in vitro model matches the in vivo regeneration in the mutated mice.

© 2022. Published by The Company of Biologists under the terms of the Creative Commons Attribution License (https://creativecommons.org/licenses/by/4.0/).

Minor points 1. Figure 1C: There is Dync1h1 signal in the het neuron, but it is located on the periphery of the cell soma- please explain if this is a true representative phenotype 2. Figure S2- It is difficult to appreciate the difference between the mice hind limb posture since the images were taken from different angles. Please provide similar images for both +/+ and +/- mice.
3. Figure 4- The image analysis is not explained, and the unit of length is not mentioned in the y axis on the graphs. An additional analysis of initiation rate quantification (% of neurons with axons/total #neurons) would be useful to understand the effect in the Dync1h1 mutant neurons. The title of the figure is misleading since it looks more like there is an enhanced growth in cultured Dync1h1 mutant neurons under basal conditions rather than a failure to respond to the conditioning injury.
4. The Methods section needs to be revised and include more detailed explanation of the image analysis techniques as well as the behavior used.

Reviewer 3

Advance summary and potential significance to field

The manuscript details the creation and characterization of conditional heterozygous dync1h1 null mice. Overall this manuscript is interesting and presents data that is of broad interest.

As outlined in the manuscript itself, this allows the characterisation of reduced dynein function in vitro and in vivo, without the possible confounding effects produced by the point mutants used historically. Consequently, the characterization of the neurological phenotypes of these conditional knockout mice when crossed with the Islet1-Cre line are likely to be of significant interest to the axonal transport and neurodegeneration communities.

In addition to this, the authors seek to link to their previous work on a possible link between molecular motors and axonal length sensing. The original hypothesis was based on a bidirectional mechanism; an anterograde signal is transported by kinesin from the cell body to the axon tip, where it activates dynein-dependent retrograde transport back to the cell body, then in turn represses the anterograde signal. This transport loop created an oscillating retrograde signal, where frequency decreased with increasing axon length. The counterintuitive prediction being that reducing levels of motors (reducing signal levels in each phase of the mechanism) leads to an increase in axon length. Conceptually, this length sensing sonar system was always very exciting, but remains difficult to prove experimentally because of the multifunctional nature of motor proteins and the unknown nature of the signal. However, the axon growth phenotypes presented by the authors in this manuscript support the hypothesis; the haploinsufficient sensory neurons do indeed grow more quickly in culture and have longer axons.

Using sciatic nerve conditioning lesions, which also cause sensory neurons to grow more quickly in culture, the authors show that this response is hidden in the Isl1-Dync1h1+/- neurons and in fact that the animals themselves were not able to recover at the same rate. This is a good illustration of the complexity of the system. The authors suggest that dync1h1 masks the effect of the conditioning lesion, however the slower recovery of the animals after injury suggests that this enhanced growth rate in vitro is not translating in vivo. I agree that the results are interesting, however I think it would be best practice to cover a few more of these nuances in the discussion (see below).

Comments for the author

I suggest the following to improve the manuscript prior to publication:
Include details of how axon length was quantified in the methods, as there are currently no details for this.
Include in the discussion some alternative explanations for the lesion results in mice, for example: the possibility that dynein haploinsufficiency is in itself a lesion, such that axons are already preconditioned for growth and this may be why they cannot respond to a second lesion; there are insufficient retrograde injury signals to stimulate growth on the haploinsufficient background.
Sometimes the ‘n’ is the number of biological repeats and sometimes it is the number of neurons analysed (e.g. Figure 4 compared to Figure 2). I assume Figure 4 is comparing the mean of means

© 2022. Published by The Company of Biologists under the terms of the Creative Commons Attribution License (https://creativecommons.org/licenses/by/4.0/).

which is why the variability in the experiment is low. It would be good to include in the supplement the opposite analysis as the ones displayed in the main figure, or some other graphical representation so that both the variance at the individual neuron level and the biological replicate level can be appreciated.
Include, in the initial characterisation of the mice, information on their postnatal growth rates and survival in comparison to controls. The islet1-Cre targets multiple tissues so it seems relevant to present this data. If life span is unaffected during the period of the experiments this can be stated.
Include in the methods details of the strain backgrounds of the mice, both the dync1h1 conditional mutant line and the Islet1-Cre line, and their maintenance.
Please include in the figure for Figure 1E a visual explanation of how the mask was generated from NFH staining. This is quite opaque at the moment as there is no mention of the NFH mask in the text or legend.
Please specify the length of the scale bar in Figure 4.

First revision

Author response to reviewers' comments

Detailed reply to Reviewer 1

1. The manuscript is sufficiently interesting and the new mouse sufficiently important that I am okay with this paper for JCS being short. I do think the authors should do a much better job with the expansive literature on how dynein contributes to axon growth, health and vitality. It seems a bit odd to interpret everything in such narrow terms as they do.

Point well-taken. We have now added more information in the **Introduction** (page 4, highlighted in yellow) with a more detailed description of the role of the DYNC1H1 protein in neurological disease, intracellular transport, and axonal growth. We also note that prolonged and near-complete Dync1h1 depletion leads to shortening of axons (Ahmad et al., 2006, *Traffic*), while on the contrary, partial Dync1h1 depletion accelerates axonal growth, consistent with our previous study (Rishal et al., 2012, *Cell Reports*).

Detailed reply to Reviewer 2

Major points:

1. The authors need to better explain why Isl1-Dync1h1+/- neurons exhibit enhanced growth in culture but at the same time show impaired recovery after nerve injury. It is also not clear how the in vitro accelerated growth relates to the in vivo phenotypes that include mild impairments in gait, proprioception and tactile sensation. As presented, one expects these two observations to be causally related. If this is not the case, it would be important to revise the text to present these two observations as independent findings that highlight the multiple function of dynein. If there is a causal relation, then innervation of muscle or skin targets could be used to see if increased growth in culture correlates with altered or impaired innervation in vivo.

The point is well-taken. We agree that it is not straightforward to link accelerated neuronal growth *in vitro* to the *in vivo* deficits reported in our study. Indeed, similar results were reported also from *Dync1h1*^{Loa} mice, comprising accelerated sensory axon growth in culture and motor and sensory deficits *in vivo*. Showing a causal relationship between these observations would indeed require examining muscle and skin innervation, among other things, but that would require comprehensive analyses in additional mouse lines with sensory versus motor neuron specific Cre drivers. Establishing such lines will require multiple generations of breeding over at least 6 months before even starting the analyses. We therefore respectfully submit that this is beyond the scope of the current manuscript. We now discuss this issue on pages 6 -7 (**Results,** highlighted in yellow) and 9 (**Discussion,** highlighted in yellow).

2. The interpretation of the results in Figure 4 is that conditioning lesion does not enhance growth in Isl1-Dync1h1+/- neurons. The author discuss that this is because growth is already

© 2022. Published by The Company of Biologists under the terms of the Creative Commons Attribution License (https://creativecommons.org/licenses/by/4.0/).

enhanced in naïve condition. Is that solely due to the length sensing mechanism previously proposed, or to the fact that the neurons are conditioned already by reduced levels of dynein heavy chain? A staining for ATF3, as a proxy for the regenerative state, in DRG sections could help address this point and determine if sensory neurons are in an active growth mode at baseline. Furthermore, analysis of nerve regeneration in sciatic nerve in vivo 1 or 3 days post injury could help understand if the in vitro model matches the in vivo regeneration in the mutated mice.

We thank the reviewer for this interesting question. To address this issue we performed sciatic nerve crush experiments in WT and *Isl1-Dync1h1*$^{+/-}$ mice and isolated L4/L5 DRG from both lesioned and contralateral naive sides on the third day after the injury. Immunostaining of DRG sections for ATF3 did not reveal any differences between naive WT and heterozygous ganglia (**Figure 5A, B**). In contrast, we saw significantly fewer DRG neuronal nuclei with upregulated ATF3 in heterozygous mice as compared to WT after injury **(Figure 5A, B)**. Taken together, these results do not support a basal conditioning effect in naive DYNC1H1+/- neurons, but they do support reduced delivery of injury signals leading to less upregulation of ATF3 in lesioned neurons. The data are presented in new figure 5, described on page 7 in the **Results (**highlighted in yellow), and discussed on page 9 (**Discussion,** highlighted in yellow).

We also prepared longitudinal sections of sciatic nerves from the same animals and verified their regenerative stage by immunostaining for the axonal regenerative marker Scg10. Quantification of Scg10 immunoreactivity 1 mm distal to the injury site revealed a significant reduction in signal intensity in heterozygous mice as compared to wild type (**Figure 7**). This is consistent with the regeneration delay found in behavioral analyses**.** These data are shown in new figure 7, described on pages 7-8 (**Results,** highlighted in yellow), and discussed on page 9 (**Discussion,** highlighted in yellow).

Minor points:

1. Figure 1C: There is Dync1h1 signal in the het neuron, but it is located on the periphery of the cell soma- please explain if this is a true representative phenotype.

We thank the reviewer for pointing this out. The figure has been updated with a more representative image.

2. Figure S2- It is difficult to appreciate the difference between the mice hind limb posture since the images were taken from different angles. Please provide similar images for both +/+ and +/- mice.

The image was replaced as requested. It now appears as **Figure S3.**

3. Figure 4- The image analysis is not explained, and the unit of length is not mentioned in the y axis on the graphs. An additional analysis of initiation rate quantification (% of neurons with axons/total #neurons) would be useful to understand the effect in the Dync1h1 mutant neurons. The title of the figure is misleading since it looks more like there is an enhanced growth in cultured Dync1h1 mutant neurons under basal conditions rather than a failure to respond to the conditioning injury.

We thank the reviewer for this comment. We changed the title of **Figure 4** to direct the reader's focus to the conditioning lesion failure in Dync1h1$^{+/-}$ mice. We added a new section on "**Axon outgrowth analysis**" on page 13 (**Materials and methods,** highlighted in yellow) detailing the parameters of **Figure 4**, and show the unit definition of the Y axis on the graph. Finally, regarding the percentage of neurons with growing axons, we added a new supplementary **Figure S5** with Panel **A** showing statistical analysis based on cell numbers and Panel **B** presenting the percentage of growing neurons in both naive and injury conditions for both genotypes. Interestingly, no difference in this parameter was observed between WT and heterozygous mice.

4. The Methods section needs to be revised and include more detailed explanation of the image analysis techniques as well as the behavior used.

© 2022. Published by The Company of Biologists under the terms of the Creative Commons Attribution License (https://creativecommons.org/licenses/by/4.0/).

We have revised the **Materials and methods** section as requested, adding more details on image acquisition and analysis in each technique. We also added new sections in **Materials and methods** that describe **Axon Outgrowth analysis** on page 13, **Immunohistochemistry** on cultured neurons and on DRG and sciatic nerve cryosections on pages 13-14. A separate section on **Quantification and statistical analysis** is now on pages 15-16.

Detailed reply to Reviewer 3

1. Include details of how axon length was quantified in the methods, as there are currently no details for this.

Please see the reply to **Reviewer 2**, **minor point 4** above.

Include in the discussion some alternative explanations for the lesion results in mice, for example: the possibility that dynein haploinsufficiency is in itself a lesion, such that axons are already preconditioned for growth and this may be why they cannot respond to a second lesion; there are insufficient retrograde injury signals to stimulate growth on the haploinsufficient background.

We appreciate the importance of these comments, which were also raised by Reviewer 2. Please see the complete response to **Major point 2** of **Reviewer 2** above.

2. Sometimes the 'n' is the number of biological repeats and sometimes it is the number of neurons analyzed (e.g. Figure 4 compared to Figure 2). I assume Figure 4 is comparing the mean of means which is why the variability in the experiment is low. It would be good to include in the supplement the opposite analysis as the ones displayed in the main figure, or some other graphical representation so that both the variance at the individual neuron level and the biological replicate level can be appreciated.

Please see the responses to **Minor points 3 and 4** of **Reviewer 2** above.

3. Include, in the initial characterization of the mice, information on their postnatal growth rates and survival in comparison to controls. The islet1-Cre targets multiple tissues so it seems relevant to present this data. If life span is unaffected during the period of the experiments this can be stated. Include in the methods details of the strain backgrounds of the mice, both the dync1h1 conditional mutant line and the Islet1-Cre line, and their maintenance.

Points well taken. We added all the requested information about characterization of the mice in a section of the **Results** entitled **"Generation of a conditional deletion allele for *Dync1h1*"** (page 5), and also on page 10 in **Materials and methods,** highlighted in yellow. We added a new **Figure S1** with details on generation of the mouse line and genotyping.

4. Please include in the figure for Figure 1E a visual explanation of how the mask was generated from NFH staining. This is quite opaque at the moment as there is no mention of the NFH mask in the text or legend.

There is no mask in Figure 1E, we apologize if the color previously used to represent NFH staining might have given this mistaken impression. NFH immunoreactivity is now depicted in blue, which hopefully clarifies the image, and additional details have been added to the figure legend. Additional details on quantification analyses of these data are now in **Materials and methods** under **"PLA labeling"** on page 12, highlighted in yellow.

Please specify the length of the scale bar in Figure 4.

Done.

© 2022. Published by The Company of Biologists under the terms of the Creative Commons Attribution License (https://creativecommons.org/licenses/by/4.0/).

Second decision letter

MS ID#: JOCES/2022/260220

MS TITLE: A conditional null allele of Dync1h1 enables targeted analyses of dynein roles in neuronal length sensing

AUTHORS: Agostina Di Pizio, Letizia Marvaldi, Marie-Christine Birling, Nataliya Okladnikov, Luc Dupuis, Mike Fainzilber, and Ida Rishal
ARTICLE TYPE: Research Article

I am happy to tell you that your manuscript has been accepted for publication in Journal of Cell Science, pending standard ethics checks and provision of an appropriate data availability section.

Reviewer 1

Advance summary and potential significance to field

Good job with the revisions.

Comments for the author

No further revisions requested.

Reviewer 2

Advance summary and potential significance to field

In this manuscript, Di Pizio et al. describe a new mouse model allowing deletion of Dync1h1 in a targeted cell type, which enables targeted studies of the role of dynein in neuronal growth. The authors show that Isl1-cre mediated deletion of dynein heavy chain (Isl1-Dync1h1+/-) in sensory neurons leads to an accelerated growth phenotype in vitro with mild impairments in gait, proprioception and tactile sensation and slightly impaired recovery from peripheral nerve injury. Overall, the authors have every nicely addressed the comments of the reviewers, with extensive revision of the text and additional experiments. The new data shows that the increased growth in vitro is not due to dynein haploinsufficiency is in itself representing an injury. The delayed regeneration in vivo in Isl1-Dync1h1+/- appears to be due to insufficient retrograde injury signals to stimulate growth on the haploinsufficient background. This study presents the characterization of very useful model for the field, which will facilitate comprehensive studies of the roles of dynein in a cell specific manner. I am okay with this paper being published in JCS.

Comments for the author

The authors have every nicely addressed the comments of the reviewers, with extensive revision of the text and additional experiments.This study presents the characterization of very useful model for the field, which will facilitate comprehensive studies of the roles of dynein in a cell specific manner. I am okay with this paper being published in JCS.

Reviewer 3

Advance summary and potential significance to field

I refer to my previous review for my summary of the paper. I thank the authors for their response to my comments and amendments to the manuscript.

Comments for the author

The statement of data availability is currently a list of software used. How is data made available?

© 2022. Published by The Company of Biologists under the terms of the Creative Commons Attribution License (https://creativecommons.org/licenses/by/4.0/).
